# A method for calculating temperature-dependent photodissociation cross sections and rates†

**DOI:** 10.1039/d1cp02162a

**Published:** 2021-07-20

**Authors:** Marco Pezzella, Sergei N. Yurchenko, Jonathan Tennyson

**Affiliations:** Department of Physics & Astronomy, University College London London WC1E 6BT UK j.tennyson@ucl.ac.uk

## Abstract

The destruction of molecules by photodissociation plays a major role in many radiation-rich environments, including the evolution of the atmospheres of exoplanets, which often exist close to UV-rich stars. Most current photodissociation calculations and databases assume *T* = 0 K, which is inadequate for hot exoplanets and stars. A method is developed for computing photodissociation spectra of diatomic molecules as a function of temperature exploiting bound state variational nuclear motion program Duo and post-processing program ExoCross. Discrete transition intensities are spread out to represent a continuous photodissociation spectrum either by Gaussian smoothing or by averaging calculations over a range of different grid sizes. Our approach is tested on four different chemical species (HCl, HF, NaCl and BeH^+^), showing its ability to reproduce photodissociation cross sections and rates computed with other approaches and experiment. The temperature dependence of photodissociation cross sections and rates is studies showing strong temperature variation of the photodissociation cross sections.

## Abstract 2

HCl potential energy curves with resulting photodissociation spectrum. The background figure (from Karen Teramura, UH IfA) shows the Kepler 78b planet with its star, and it is an example of a planet immersed in a stellar radiation field.

## Graphical abstract

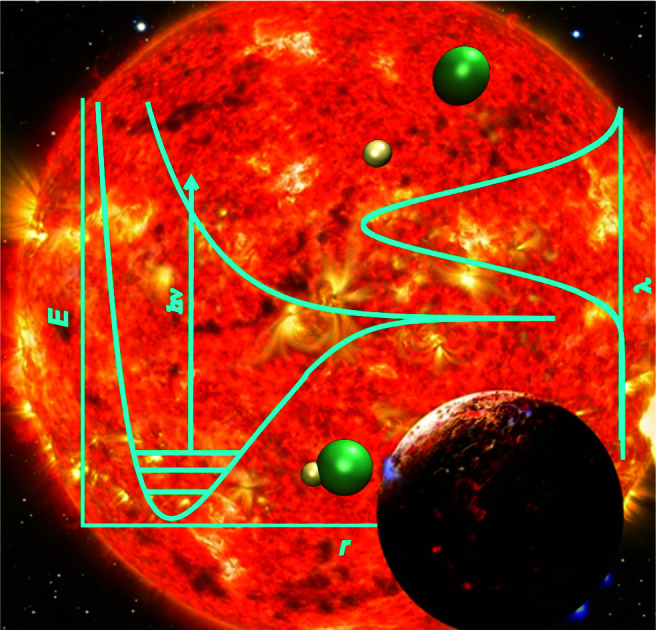

## 1 Introduction

Photochemistry substantially impacts the atmospheric composition of planets and exoplanets with consequences for the chemical compositions, radiative transfer, thermal structure, and dynamics of the atmospheres. This is particularly true for the many exoplanets that have been discovered orbiting near their host stars as these planets exists in UV-rich environments.^1–4^ Of course, it is exactly the planets which experience high UV fluxes where the molecules are also hot and hence vibrationally and rotationally excited. Modelling and understanding the atmospheres of such planets therefore requires temperature-dependent photodissociation cross sections. Measurements of photodissociation cross sections of molecules at higher temperatures have been performed^1^ but these studies struggle to reach the temperatures needed for the top of hot atmospheres (*T* > 1000 K).

Current state-of-the-art for calculation of photodissociation cross sections for astronomical studies often use simplified (harmonic) ground state wavefunctions.^5^ This model is appropriate for the cold molecules such as those found in the interstellar medium (ISM) but inadequate for hot environments such as the atmospheres of exoplanets. Here we present a novel methodology aimed at resolving this problem. Similarly, there are standard databases of photodissociation cross sections studies of the ISM^6,7^ but these only contain data for molecules at interstellar temperatures, often assumed to be 0 K.

There is a long history of theoretical treatments of photodissociation,^8^ but it is only recently that cross section calculations have begun to seriously consider the effects of temperature^9^ and even then the effects of rotational excitation appears to have been largely ignored.

The ExoMol project was designed to produce comprehensive line lists of bound–bound transitions for molecules in hot atmospheres.^10^ The ExoMol database provides such line lists for a large range of molecules deemed to be important in exoplanets and elsewhere.^11^ As part of the ExoMol project a series of nuclear motion codes have been developed or enhanced to give results which are both comprehensive and accurate.^12^ Here we concentrate on one of the programs, Duo.^13^ Duo solves the bound–bound diatomic nuclear motion problem by explicit solution of the nuclear motion Schrödinger equation and allows for treatment of spin–orbit and other coupling effects^14^ as well avoided and allowed curve crossings.^15^ The treatment we propose here is based on extending Duo to treat the bound–free problem of photodissociation. This will allow temperature effects to be fully captured; our procedure can also provide the requisite data for modelling the effect of photodissociation in environments where non-local thermodynamic equilibrium (non-LTE) is important. Such data are not obtainable with current experimental procedures. We note that photodissociation itself is likely to prove to be a major driver of non-LTE regimes.

Duo has already been adapted for the study of continuum (free–free) states within an R-matrix formalism with a particular focus on the treatment of ultracold collisions.^16^ Here we propose a rather more radical approach where the continuum is only modelled using a (finite) inner region and photodissociation cross sections or rates are extracted from the results. This approach avoids the need for computationally expensive treatment of the long-range wavefunctions making studies over many rotational states easy and fast. The approach allows the full photoabsorption problem, *i.e.*, bound–bound and bound–free transitions, to be treated within a single formalism and on an equal footing.

## 2 Theory and methods

### 2.1 Theoretical background

Our treatment of the diatomic photoabsorption problem, which includes both bound–bound transitions and bound–free (photodissociation) transitions is based on the diatomic code Duo.^13^ Duo uses a variational procedure to find solutions to the multistate rovibronic nuclear motion problem allowing for treatment of spin–orbit and other couplings.^14^ Duo has been extensively used to provide accurate line lists for challenging bound–bound problems.^17–22^ The present implementation uses Duo to solve the nuclear motion Schrödinger equation and then post-processing program ExoCross^23^ to produce photodissociation cross sections and rates, and, optionally, to distinguish between bound–bound and bound–free transitions. Duo initially uses a grid basis set to solve the rotationless one-dimensional Schrödinger equation separately for each electronic state *Γ*:1
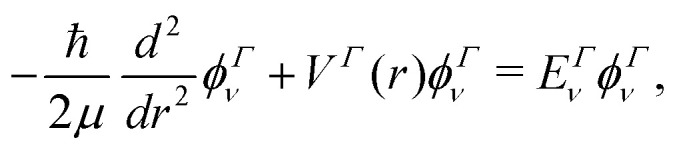
producing a set of vibrational wavefunctions *ϕ*^Γ^_*ν*_. Here *V*^Γ^(*r*) is the corresponding potential energy curve (PEC), *μ* is the reduced mass of the molecule, *r* the interatomic distance, and *ν* the vibrational quantum number. In the present implementation solutions can be obtained using a grid based on a sinc DVR (discrete variable representation)^24^ or on Lobatto shape functions^16,25^ or a five-point finite differences to derive the kinetic energy operator.

The transition dipole moment curves, 
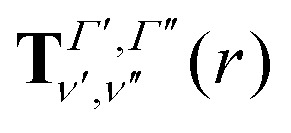
 between two vibronic states 
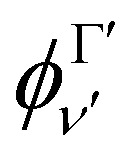
 and 
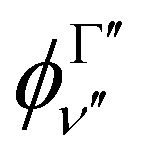
 are expressed as:2
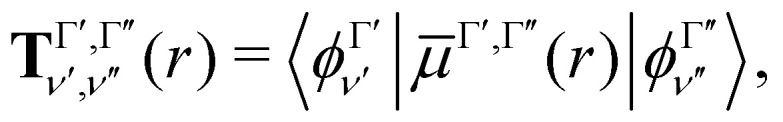
with 
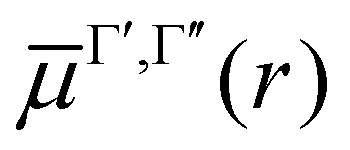
 as the electronic dipole moment vector and are used for evaluating the intensity of the transitions.

The vibrational wavefunction are then combined with suitable angular functions in a Hund's case a representation, to provide a basis set for each total angular momentum quantum number, *J*. These basis sets are used to solve the full Hamiltonian, that couples rovibronic states *ψ*^*J*^_*i*_ belonging to different electronic states and different values of the angular momenta *J*. Additional terms can be added to take into account non-adiabatic couplings between curves,^15^ and allow for spin–orbit and other similar couplings. Only transitions *f* ← *i* that obey the electric dipole moment selection rules parity changes and

3 Δ*J* = *J*_*f*_ − *J*_*i*_ = 0, ±1

are allowed.

The intensity *I*^*f*←*i*^ of a given bound–bound transition also depends on the rotational number of the initial (*J*_*i*_) and final (*J*_*f*_) states and on the temperature (*T*):4
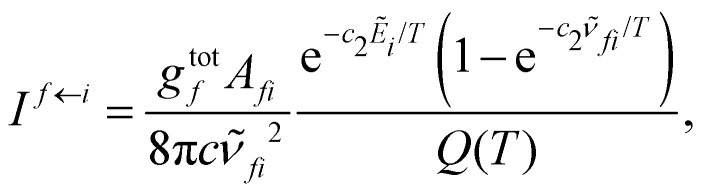
where *A*_*fi*_ is the Einstein-A coefficient (s^−1^) computed using the Duo rovibronic wavefunctions |*ψ*^*J*^_*i*_〉, *ν̃*_*fi*_ is the transition wavenumber (cm^−1^), 
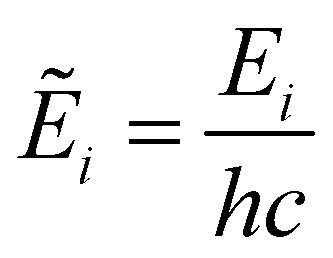
 is the term value (cm^−1^), 
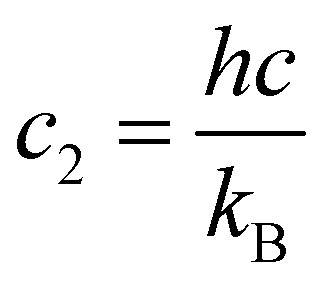
 is the second radiations constant (cm K^−1^); *h* is the Planck constant, *c* the speed of light, *k*_B_ the Boltzmann constant; *g*^tot^_*i*_ is the total nuclear statistical weight factor*g*^tot^_*i*_ = *g*^ns^_*i*_(2*J*_*i*_ + 1),where *g*^ns^_*i*_ is nuclear spin statistical weight; *Q*(*T*) is the partition function defined as a sum over bound states5
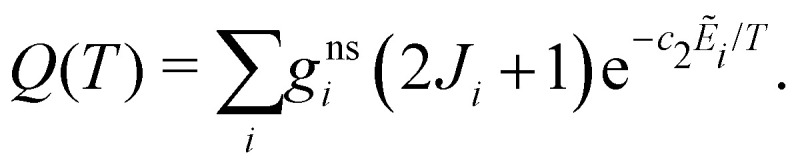


The intensity is the integral of the cross section *σ*_*fi*_ over an absorption line:6
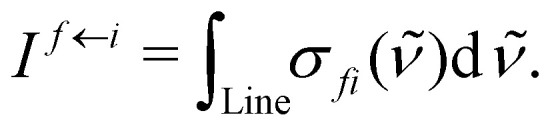


By introducing a line profile *f*_*ν̃*_*fi*__(*ν̃*), *σ*_*fi*_(*ν̃*) can be defined as

7 *σ*_*fi*_(*ν̃*) = *I*^*f*←*i*^*f*_*ν̃*_*fi*__(*ν̃*),

where *f*_ν̃_*fi*__(*ν̃*) is an integrable function which is normalized to unity.

The bound–free photodissociation process is characterized by the excitation from a bound electronic state, usually the electronic ground state, to an unbound rovibronic level of an excited state. The radial wavefunctions of these dissociative states are described by a sinusoidal wavefunction at the asymptotic limit. Here we adapt eqn (4) to cover both bound–bound and bound–free processes. We note that the self-absorption term in eqn (4), given by −e^−*c*_2_*ν̃*_*fi*_/*T*^, is probably not needed for bound–free transitions^26^ but in practice will be negligible for the short wavelength processes considered here. A future refinement will be to remove this term for bound–free processes.

### 2.2 Continuum cross section calculations

**Fig. 1:**
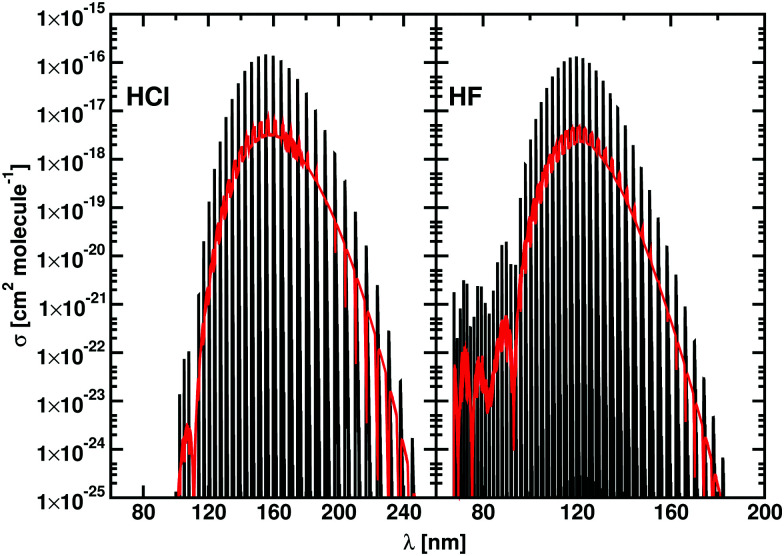
Cross sections generated using a Gaussian with HWHM of 10 cm^−1^ for HCl and HF from a single run in Duo (black) and with the stabilization method (red). Our calculations are performed at 100 K.

Duo is designed to provide discrete solutions for bound electronic systems of diatomics. Here we present a robust approach to use Duo for computing temperature-dependent photo-dissociation spectra of diatomics representing bound–free transitions. To this end, a coupled set of Schrödinger equations for the system containing bound and unbound PECs is solved on the basis of bound vibrational functions *ϕ*^Γ^ from eqn (1). The discrete eigenvalues *Ẽ*_*i*_ and eigenfunctions *ψ*^*J*^_*i*_ are then used to generate line intensities *via*eqn (4). This gives a photodissociation spectrum which is represented by clusters of discrete, high intensity lines, separated by regions where there is no intensity, see Fig. 1. To recover the continuum nature of the spectrum we apply a smoothing function to the cross sections, computed using the ExoCross program.^23^

We have tested two smoothing functions included into the SciPy^27^ package. The first method consists in interpolating the spectrum with knots equally distributed along the wavelengths. The second method consist of applying a normalized Gaussian smoothing function to each grid point. The Gaussian line profile is given by:^28^8
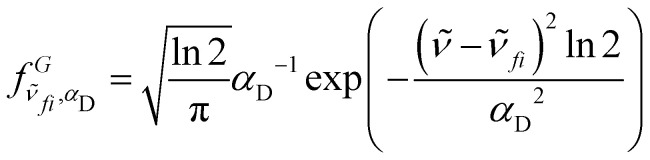
where *ν̃*_*fi*_ is the line centre and *α*_D_ is the Gaussian half width at half maximum (HWHM).

The HWHM of the Gaussian line profile is used for regulating the cross section height. Optimal values for the HWHMs depend strongly on the molecule under analysis, with values between 2000 cm^−1^ and 3000 cm^−1^ for hydrogen halides (see Section 3.1), and 75 cm^−1^ in case of high temperature cross sections for BeH^+^ (Section 3.3). The appropriate HWHM depends on several factors including the size and density of the grid used, which determines the number of discrete transitions given by Duo, and the temperature as the large number of lines at elevated temperatures leads to dense spectra making further smoothing beyond the initial 10 cm^−1^ broadening unnecessary. Both methods are designed to conserve the integrated intensity and the photodissociation rates with respect to the initial transition intensities.

Some excited potentials support a few bound vibrational states. These states manifest themselves in a photoabsorption spectrum as a series of bound–bound transitions at longer wavelengths than the transitions to those states which are responsible for photodissociation. It is therefore important to be able distinguishing bound–free transitions from bound–bound ones. Here we adopt an approach that has some similarities to the stabilization method of Taylor and co-workers.^29–31^ In our case, the Duo calculations are repeated using different grid sizes which are varied by a few tenths of an Å. Each calculation results in temperature-depended cross sections, which are then averaged to produce our final photodissociation cross sections. The individual cross sections are obtained using a Gaussian line profile of HWHM = 10 cm^−1^. These repeated calculations smear out the bound–free transitions, possibly leading to results that are more easily smoothed to give continuum cross sections, but leave bound–bound transitions in the same place allowing them to be readily identified. Photodisocciation cross sections generated using the stabilization method are illustrated in Fig. 1.

If one only wants photodissociation cross sections or rates, bound–bound contributions need to be identified and discounted. Identification of bound–bound transitions is facilitated by using the stabilization method as they always occur with the same transition frequency when the box size is varied. The photodissociation spectrum can be recovered by calculating the overall photoabsorption spectrum and then subtracting the bound–bound transitions contribution. An alternative approach consists of summing photodissociation cross sections evaluated for each single state in turn excluding the bound contributions. Results of these two methods are compared in Section 3.3.

### 2.3 Photodissociation rates

For many purposes photodissociation rates are used instead of cross sections; for example, rates are used for modelling the abundance and the evolution of species in space.^32,33^ The rates provide a useful quantity to test the validity of our approach for the molecules we study. The photodissociation rate *k* of a molecule dissociated by a field with a flux *F*(*ν̃*) between the wavelengths *ν̃*_1_ and *ν̃*_2_ is expressed as:9
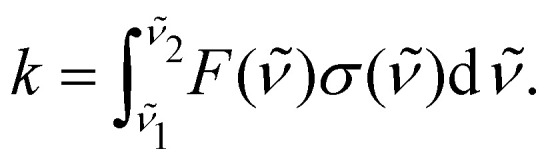


There are several standard fluxes used to produce appropriate rates. Here we concentrate on the flux appropriate for the interstellar medium (ISM) since this is widely used by the databases to which we want to compare. Future work will consider a variety of stellar fluxes. Photodissociation rates are calculated using the interstellar radiation field (ISRF).^34–36^ The ISRF has been fitted to an analytical expression for wavelengths between 91.2 nm and 200 nm, and was expressed as:

10 *F*(*λ*) = 3.2028 × 10^13^*λ*^−3^ − 5.1542 × 10^15^*λ*^−4^ + 2.0546 × 10^17^*λ*^−5^

where *λ* is the wavelength in nm and it was later extended to 2000 nm using the expression:

11 *F*(*λ*) = 3.67 × 10^4^*λ*^0.7^.

## 3 Results

Our approach is tested for three different system types. The first consists of the A^1^Π ← X^1^Σ^+^ photodissociation from the vibrational ground state for two hydrogen halides: HCl and HF. The second system is NaCl and its photodissociation as a function of temperature. With the third system, BeH^+^, we compare the results from our method with recently published calculations. All calculations are carried using a 8 core Intel local machine with 32 GB of RAM using available potential energy and transition dipole curves.

### 3.1 HCl and HF

HCl and HF are characterized by the presence of a repulsive A^1^Π excited state. The A^1^Π← X^1^Σ^+^ electronic transition leads to the immediate photodissociation into two neutral fragments H(^2^S) + X(^2^P), where X is F or Cl. Photodissociation arising from these electronic transitions was chosen as an initial test of our methodology.

For HCl we used the X^1^Σ^+^ and the A^1^Π potentials taken from Alexander *et al.*^37^ and the A^1^Π ← X^1^Σ^+^ transition dipole moment from Givertz and Balint-Kurti.^38^ For HF the X^1^Σ^+^, A^1^Π potentials and the A^1^Π ← X^1^Σ^+^ transition dipole moments of HF are taken from Brown and Balint-Kurti.^39^ For both molecules, vibrational wavefunctions were built for *J* = 0, between 0.5 Å and 3.0 Å. Transitions from the vibrational ground state of the X^1^Σ^+^ state to the A^1^Π state were considered for a temperature of *T* = 100 K, which required *J* up to 16 for HCl and up to 11 for HF. Calculations were also performed with the time-dependent Schrödinger code PHOTO, developed by Balint-Kurti *et al.*,^40^ which only considers states with *J* = 0 and is hence useful for a low temperature comparison.

Fig. 1 presents photodissociation cross sections of HCl and HF computed using the stabilization approach for *T* = 100 K. The black lines in Fig. 1 show the intensity of the discretised transitions to the continuum obtained running a single calculation with Duo and ExoCross on a grid of 2001 points ranging from 0.5 Å to 3.00 Å. These spectra of HCl and HF consist in clusters of discrete lines, characterized by high values of *σ*(*ν̃*), separated by regions where *σ*(*ν̃*) = 0. The red curves in Fig. 1 show the spectra calculated with the stabilization method extending the original grid from 2.50 Å to 2.60 Å with steps of 0.001 Å, obtained by averaging 100 individual cross sections, each computed using the Gaussian line profile with HWHM = 10 cm^−1^. The final spectrum consists of a discrete spectrum overlapped to a continuum background. The magnitude of the cross section for the discrete spectrum obtained with this procedure is 20 times smaller the peaks in the black curves of Fig. 1.

**Fig. 2:**
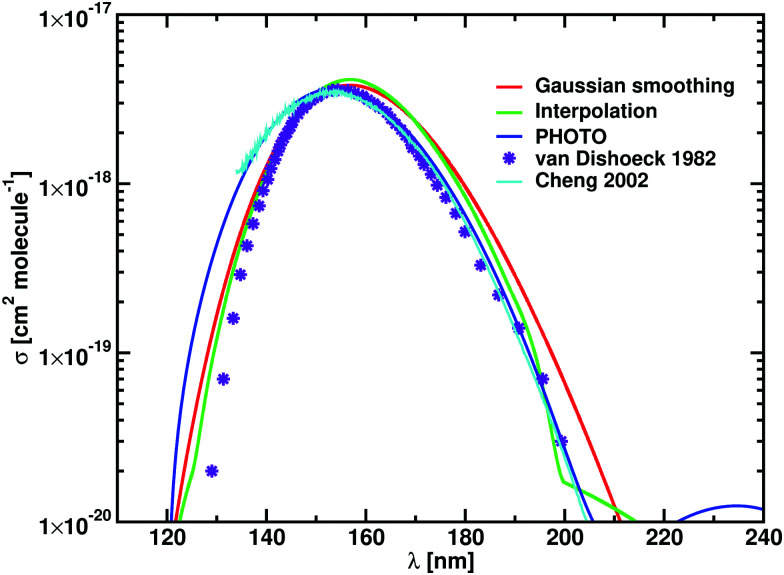
Photodissociation cross sections of HCl, numerical values are in Table 2. Results from Duo and ExoCross with the Gaussian smoothing are plotted in red, with the interpolation in green. Calculations carried with PHOTO are blue, the data from van Dishoeck *et al.* in violet,^44^ and the experimental data in cyan.^43^ Our calculations are performed at 100 K.

Now we apply the Gaussian smoothing method to produce the *T* = 100 K photodissociation cross sections of HCl as described above. These are compared to experimental results^41–43^ and to the results from PHOTO^40^ with the numerical results are reported in Table 2 and plotted in Fig. 2. The Gaussian smoothing model with HWHM of 1800 cm^−1^ produces cross sections with *σ*_max_ = 3.55 × 10^−18^ cm^2^ molecule^−1^, while the interpolation scheme (with knots at every 5 nm) leads to a higher value of *σ*_max_ = 4.13 × 10^−18^ cm^2^ molecule^−1^, but within the upper limits reported by Inn.^41^ The peak position (*λ*_max_) is overestimated by 3 Å with respect to PHOTO and experiments. Our photodissociation rates calculated for ISRF before and after smoothing give the same value of *k* = 2.29 × 10^−10^ s^−1^ at *T* = 100 K. The photodissociation rates differ between our model and PHOTO only by the 4%.

The experimental HCl photodissociation cross section shows an asymmetry at short wavelengths, see Fig. 2, due to a non-adiabatic coupling between the A^1^Π and the C^1^Π states.^43^ The maximum of the cross section is found at *λ*_max_ = 153.0 nm, with *σ*_max_ = 3.53 × 10^−18^ cm^2^ molecule^−1^. The computer model of van Dishoeck *et al.*^44^ overestimates *λ*_max_ by 2.3 nm with respect to the experiments and estimates a photodissociation rate of *k* = 2.1 × 10^−10^ s^−1^. The time dependent Schrödinger code PHOTO estimates the photodissociation rate of *k* = 2.188 × 10^−10^ s^−1^.

Three different types of basis functions were tested in Duo, corresponding to Sinc DVR, Lobatto and the 5 point finite differences; Table 1 shows the corresponding values of *λ*_max_, *σ*_max_ as well as of the integrated intensity *I*. The calculations were performed on a grid of 601 points. The Lobatto wavefunctions lead to the largest difference from the other methods, especially in case of HF. For this molecule we observe a shift of the maximum cross section peak *λ*_max_ of 2.08 nm and an overestimation of the cross section maximum of the order of 18%. All further calculations in this work are performed using the sync DVR basis function.

**Table 1:** Comparison of results obtained using three different basis functions, corresponding to the sinc DVR, Lobatto wavefunctions, and five-point finite differences methods, for HCl and HF. Calculations are performed on 601 point grid at 100 K for an interatomic distance between 0.5 and 3 Å. The properties considered are the wavelength of the cross section peak (*λ*_max_, nm), the cross section maximum (*σ*_max_, cm^2^ molecule^−1^), the integrated intensity (*I*, cm molecule^−1^), and the partition function (*Q*) at *T*= 100 K

	Sinc DVR	Lobatto	5 pt differences
HCl			
*λ*_max_	155.21	154.29	155.37
*σ*_max_	1.47 × 10^−16^	1.72 × 10^−16^	1.47 × 10^−16^
*I*	4.84 × 10^−14^	4.81 × 10^−14^	4.84 × 10^−14^
*Q*	5.64	5.68	5.64

HF			
*λ*_max_	119.90	121.98	119.91
*σ*_max_	1.33 × 10^−16^	1.46 × 10^−16^	1.33 × 10^−16^
*I*	4.30 × 10^−14^	4.19 × 10^−14^	4.30 × 10^−14^
*Q*	15.31	15.46	15.31

**Table 2:** HCl photodissociation cross sections and rates for the A^1^Π ← X^1^Σ^+^ electronic transition, using different smoothing methods. *λ*_max_ is in nm, *σ*_max_ in cm^2^ molecule^−1^ and *k* in s^−1^

Model	*λ*_max_	*σ*_max_	*k*
Experimental^41^	153.00 ± 0.05	3.82 ± 0.38 × 10^−18^	—
Experimental^42^	153.90 ± 0.05	3.28 ± 0.49 × 10^−18^	—
Experimental^43^	153.90 ± 0.02	3.53 ± 0.18 × 10^−18^	—
van Dishoeck *et al.*^44^	154.4	3.5 × 10^−18^	2.1 × 10^−10^
PHOTO	153.63	3.644 × 10^−18^	2.188 × 10^−10^
Raw data	155.21	1.472 × 10^−16^	2.289 × 10^−10^
Gaussian smooth	156.76	3.553 × 10^−18^	2.290 × 10^−10^
Interpolation	156.88	4.135 × 10^−18^	2.290 × 10^−10^

There is a weak dependence of the cross section on the number of grid points. Increasing the size from 251 to 4001 points, for both HCl and HF, there is an increase of 0.6% in *σ*_max_ and of 0.3% in *λ*_max_.

**Fig. 3:**
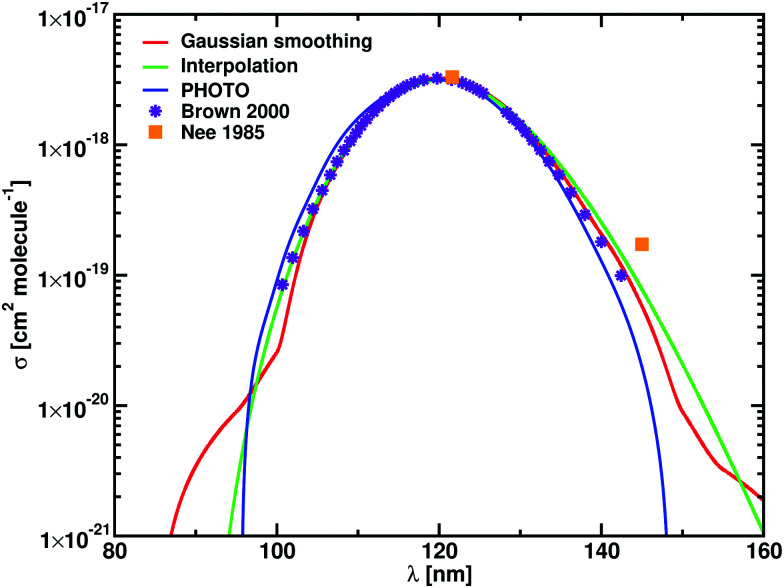
Cross sections for HF for computational models presented in Table 3. Gaussian smoothing results are in red, results of interpolation in green, and calculation performed with PHOTO in blue; the calculations of Brown and Balint-Kurti^39^ are in violet. The Gaussian smoothing model is sensitive to the high energy tail shown in Fig. 1.

The theoretical and experimental photodissociation cross sections of HF are compared in Fig. 3. The shape of the HF photodissociation cross section is symmetric, with measured values of *λ*_max_ varying between 119.8 and 121.7 nm.^45,46^*σ*_max_ also varies greatly between the two experiments, from 6 × 10^−18^ cm^2^ molecule^−1^ given by Hitchcock *et al.*^45^ to 3.3 × 10^−18^ cm^2^ molecule^−1^ given by Nee *et al.*^46^ Previous computational results^39^ agree with the results of Nee *et al.*^46^

Our *λ*_max_ for HF at *T* = 100 K agrees with Hitchcock *et al.*^45^ The spectrum from Duo and ExoCross show a high energy tail at 90 nm (see Fig. 3), that is reproduced in the Gaussian smoothing model with a HWHM of 2700 cm^−1^. Our photodissociation rates (1.173 × 10^−10^ s^−1^) agree within an uncertainty of 4% with the rates calculated by Brown and Balint-Kurti^39^ and our calculations using PHOTO. Table 3 compares calculated and experimental values of *λ*_max_, *σ*_max_ and *k*.

**Table 3:** HF photodissociation cross sections and rates for the A^1^Π ← X^1^Σ^+^ electronic transition, using different smoothing methods (*T* = 100 K) compared to experiment and PHOTO. It should be noted that PHOTO assumes *T* = 0 K. The temperatures for experimental data were not specified and were assumed to be 300 K. *λ*_max_ is in nm, *σ*_max_ in cm^2^ molecule^−1^ and *k* in s^−1^

Model	*λ*_max_	*σ*_max_	*k*
Experiment^45^	119.81 ± 0.06	6 ± 1 × 10^−18^	—
Experiment^46^	121.7 ± 0.3	3.3 ± 0.3 × 10^−18^	—
Brown^39^	121.6	3.10 × 10^−18^	1.148 × 10^−10^
PHOTO	119.479	3.156 × 10^−18^	1.129 × 10^−10^
Raw data	119.800	1.330 × 10^−16^	1.173 × 10^−10^
Gaussian smoothing	120.197	3.128 × 10^−18^	1.173 × 10^−10^
Interpolation	120.006	3.254 × 10^−18^	1.173 × 10^−10^

**Fig. 4:**
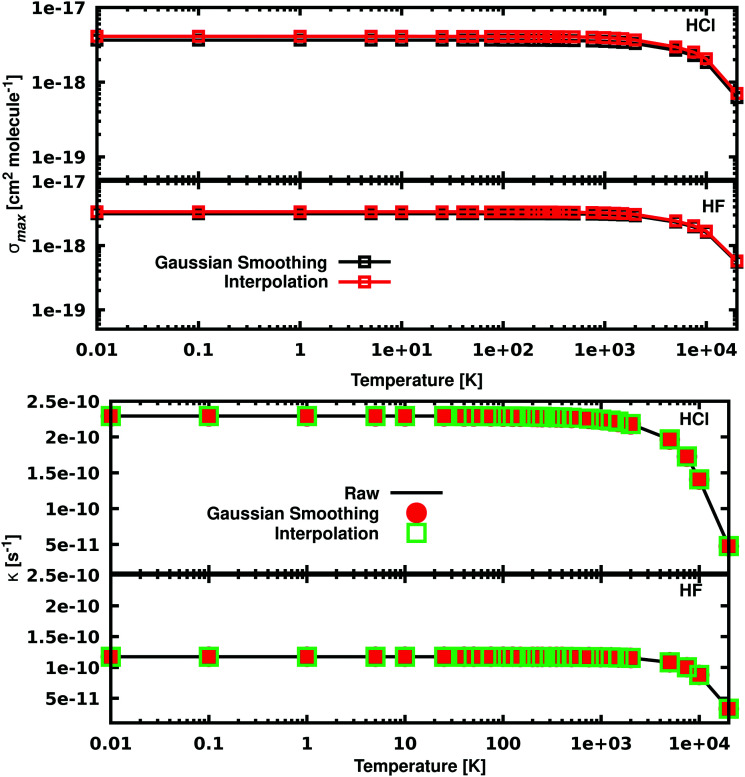
Temperature dependence of the cross sections maximum, top graph, and of the photodissociation rates, bottom graph, for HCl and HF. Cross sections from the Gaussian smoothing (black) and from the interpolation (red) show the same temperature dependence with *σ*_max_ slowly decreasing as function of temperature. The photodissociation rates decrease with the temperature as well, with all the models, the raw, unsmoothed data (black lines), the red dots (red circles) and the interpolation (green squares), giving the same results.

The temperature dependence of *σ*_max_ and *k* is reported in Fig. 4. For temperatures below 1000 K, *σ*_max_ is constant and it starts decreasing at higher temperatures. In the case of HCl, at 0.01 K *σ*_max_ = 3.53 × 10^−18^ cm^2^ molecule^−1^ and decreases to *σ*_max_ = 4.77 × 10^−19^ cm^2^ molecule^−1^ at 20 000 K for the Gaussian smoothing model. The same trend is observed for HF, with *σ*_max_ = 3.13 × 10^−18^ at 0.01 K and *σ*_max_ = 5.50 × 10^−19^ at 20 000 K. The bottom part of Fig. 4 shows that photodissociation rates for HCl and HF with the expected decrease when temperature increases. The figure shows near perfect agreement, within the 0.2%, between the rates calculated using the raw, unsmoothed data from ExoCross and the two smoothing models.

**Fig. 5:**
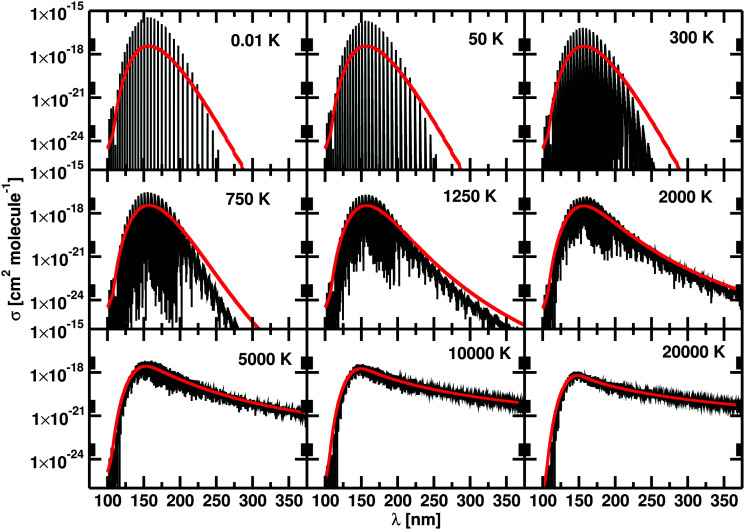
Unsmoothed (black) and smoothed (red) cross sections of HCl at different temperatures. With the increase of temperature from 0.01 K to 20 000 K, the filling between lines becomes smaller up to the point that a continuous spectrum is recovered. The unsmoothed and smoothed spectra coincide from 2000 K. The number of accessible rovibrational states increase with the temperature, reducing the difference between the unsmoothed and smoothed data. This effect is evident at long wavelengths, above 225 nm.

The temperature increase leads to an increase of population of the excited (bound) rovibrational states of the lower, ground electronic state. As a consequence, the photodissociation spectrum has a more dense character forming featureless spectral background even without extra smoothing applied. Fig. 5 shows the unsmoothed and Gaussian smoothed cross section for HCl at different temperatures: the two of them present the same shape for temperatures equal to or higher than 2000 K.

### 3.2 NaCl

Experimental photodissociation cross sections for NaCl have been reported for *T* = 300 K Na ^+^ + Cl^−^ dissociation products,^47^ and at *T* = 1123 K for the neutral dissociation products.^48^ The photodissociation spectrum above 200 nm comprises two contributions from the A^1^Π ← X^1^Σ^+^ and the B^1^Σ^+^ ←X^1^Σ^+^ transitions. At low temperatures, both bands form distinct, observable structures, as shown in Silver *et al.*,^47^ while they merge at higher temperatures: the cross section at 1123 K shows a maximum at *λ* = 236 nm with *σ* = 3.5 ± 0.3 × 10^−17^ cm^2^.^48^

Our calculations are performed using the potential energy curves, dipole moments and couplings from the ExoMol study of Barton *et al.*,^49^ where they were used to calculate the X^1^Σ^+^ state rovibrational spectrum. As part of the current work, we have produced components required for modelling the electronic A–X and B–X spectra using the MRCI/aug-cc-pVQZ *ab initio* level of theory as implemented in MOLPRO.^50^ This includes the PECs X^1^Σ^+^, A^1^Π and B^1^Σ^+^, the transition dipole moment curves A–X and B–X and an electronic angular momentum coupling curve between the A and B state. The active space was selected to be (10, 4, 4, 0) with (4, 2, 2, 0) closed orbitals.

**Fig. 6:**
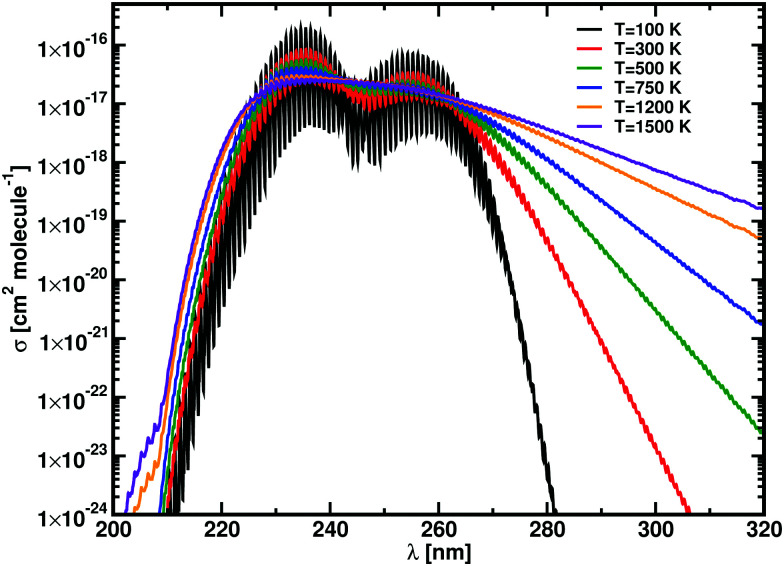
NaCl cross sections calculated at different temperatures, *T* = 100 K black, *T* = 300 K red, *T* = 500 K green, *T* = 750 K blue, *T* = 1200 K orange and *T* = 1500 K violet. The A^1^Π ← X^1^Σ^+^ contribution and the vibrational structures disappear with the increase of temperature.

**Fig. 7:**
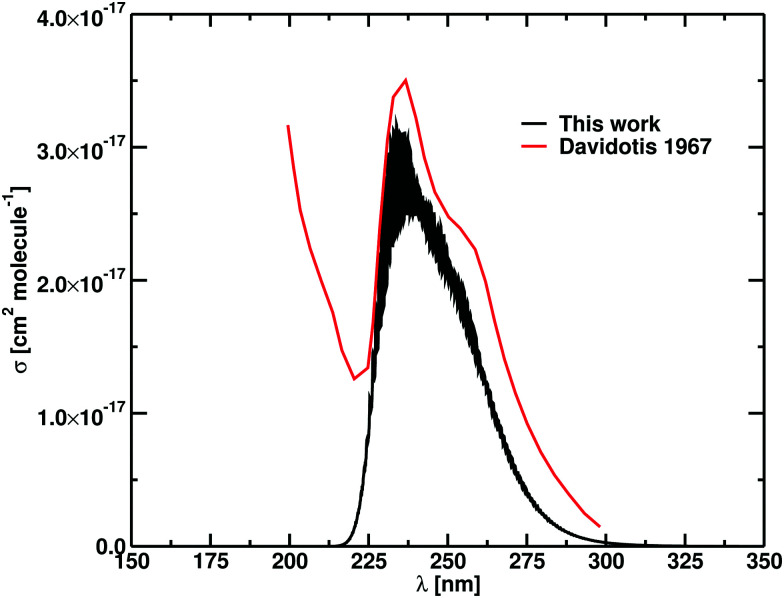
Photodissociation cross section of NaCl at *T* = 1123 K. Results for our calculations after Gaussian smoothing (HWHM = 100 cm^−1^) is in black, while the experimental values from Davidovits and Brodhead^48^ are plotted in red.

For temperatures below 1000 K, transitions up to *J* = 100 are used; this is extended to *J* = 291 for higher temperatures. A 1001 point grid with points between 1.5 Å and 5 Å is used, selecting 60 vibrations states for the X^1^Σ^+^ states, 150 for the A^1^Π and 120 for the B^1^Σ^+^. The electronic angular momentum coupling curve *L̂*_*x*_ is considered between the X^1^Σ^+^ and the A^1^Π states. The calculated photodissociation cross sections of NaCl for different temperatures are plotted in Fig. 6 with the corresponding *λ*_max_ and *σ*_max_ values tabulated in Table 4. A direct comparison between our calculations and experimental cross sections of NaCl at 1123 K are shown in Fig. 7. Our calculations reproduce the two peak structure in the NaCl cross sections and the disappearance of the lower energy peak at high temperatures. The photodissociation band A^1^Π ← X^1^Σ^+^ is very distinct at *T* = 100 K, *T* = 300 K, *T* = 500 K, turning a into shoulder at *T* = 750 K. It is submerged by the B^1^Σ^+^ ← X^1^Σ^+^ band at higher temperatures. For the analysis presented in Table 4, their contributions for *T* ≥ 1200 K were separated as indicated by an asterisk. The B^1^Σ^+^ ← X^1^Σ^+^ transition shows a blue shift of 1.71 nm and a decrease of the cross section from 19.1 × 10^−16^ to 2.67 × 10^−17^ over the 100–1500 K interval. A direct comparison between the cross sections at 300 K of Silver *et al.*^47^ is not possible, due to the different final states. The photodissociation rate for the ISRF field shows almost no temperature dependence, passing from 9.24 × 10^−10^ s^−1^ at *T* = 100 K to 9.26 × 10^−10^ s^−1^ at *T* = 1500 K.

**Table 4:** NaCl cross section and rates at different temperatures. Contributions deriving from the B1Σ^+^ ← X^1^Σ^+^ and A^1^Π ← X^1^Σ^+^ transitions, are separated. For *T* > 1200 K, the low energy cross section is submerged by the high energy one; these cases are evidenced by an asterisk. The temperature is in Kelvin, *λ*^B←X^_max_ and *λ*^A←X^_max_ are in nm, *σ*^B←X^_max_ and *σ*^A←X^_max_ are cm^2^ molecule^−1^, and *k* in s^−1^

Temperature [K]	*λ* ^B←X^_max_ [nm]	*σ* ^B←X^_max_ [cm^2^ molecule^−1^]	*λ* ^A←X^_max_ [nm]	*σ* ^A←X^_max_ [cm^2^ molecule^−1^]	*k* [s^−1^]
100	235.53	1.91 × 10^−16^	254.29	7.16 × 10^−17^	9.23 × 10^−10^
300	235.53	8.51 × 10^−17^	254.29	3.38 × 10^−17^	9.23 × 10^−10^
500	235.53	5.62 × 10^−17^	254.31	2.46 × 10^−17^	9.23 × 10^−10^
750	235.53	4.13 × 10^− 17^	254.33	2.15 × 10^− 18^	9.24 × 10^− 10^
1200*	233.82	3.05 × 10^−17^	254.32	1.26 × 10^− 17^	9.26 × 10^− 9^
1500*	233.82	2.67 × 10^−17^	254.33	1.07 × 10^−17^	9.26 × 10^−10^

The experimental *T* = 1123 K cross section by Davidovits and Brodhead^48^ (see Fig. 7) shows a feature from the C^1^Π ← X^1^Σ^+^ band at shorter wavelengths, which is not present in our model. The smoothed curve is characterized by *λ*_max_ = 233.82 nm *versus* the experimental value of *λ*_max_ = 236 nm with a cross section of 3.17 × 10^−17^ cm^2^ molecule^−1^, within the uncertainty range of the experimental results.

### 3.3 BeH^+^

Beryllium is the lightest stable nuclide not synthesized in the Big Bang, and it is a probe to study the early Universe structure and evolution.^51,52^ The hydrogenated species BeH^+^ finds application in plasma and nuclear physics.^53,54^ The potential energy curves, transition dipole moments, spectroscopic data and photodissociation cross sections of BeH^+^ covering the X^1^Σ^+^, A^1^Σ^+^, B^1^Π, C^1^Σ^+^ states at 1800 K, 4500 K, 10 000 K and 20 000 K have been reported by Xu *et al.*^55^ and Yang *et al.*^56^ The photodissociation spectrum of BeH^+^ comprises two peaks, the first from the A^1^Σ^+^, B^1^Π ← X^1^Σ^+^ bands that correlates with the Be^+^(2p) + H(1s), and the second one from the C^1^Σ^+^ ← X^1^Σ^+^ band, which correlates with the Be(2s^2^) + H^+^ asymptote. All the three excited electronic states support bound vibrational states: 23 for A^1^Σ^+^, 13 for B^1^Π, and 7 for C^1^Σ^+^.

The coupled system of Schrödinger equations is solved using Duo with PECs of BeH^+^ from Xu *et al.*^55^ on a grid of 4001 points between 0.5 and 6.0 Å, evaluating 400 vibrational states (both bound and unbound) for X^1^Σ^+^, 393 for A^1^Σ^+^, 391 for B^1^Π, and 381 for C^1^Σ^+^, imposing *E*_max_ = 998 841 cm^−1^. The X^1^Σ^+^ state can hold 20 bound vibrational states with the rotational excitations ranging up to *J*_max_ = 56. The photodissociation intensities were computed using Xu *et al.*'s transition dipole moment curves. For all temperatures, *J*_max_ = 32 was used chosen as to maximize the agreement with the calculations of Yang *et al.*^56^

Fig. 9 shows an example of how the photodissociation cross section of BeH^+^ is recovered from the photoabsoption spectrum on top of the PECs involved. The bound–free transitions are given by dashed arrows, while the bound–bound transitions are depicted by the straight arrows. The inset in the figure shows the photoabsorption spectrum calculated at *T* = 1800 K. An estimate of the photodissociation cross section directly excluding the bound–bound contributions leads to the same trend as the previous method. Fig. 10 show the outcome of the two methods compared with the results from Yang *et al.*^56^ in Panel A, the absolute difference between our two approaches is shown in Panel B. The greatest differences encountered at the boundaries, while the relative difference between the two methods is of the order of the 5 × 10^−4^, making them equivalent for our purposes.

**Fig. 8:**
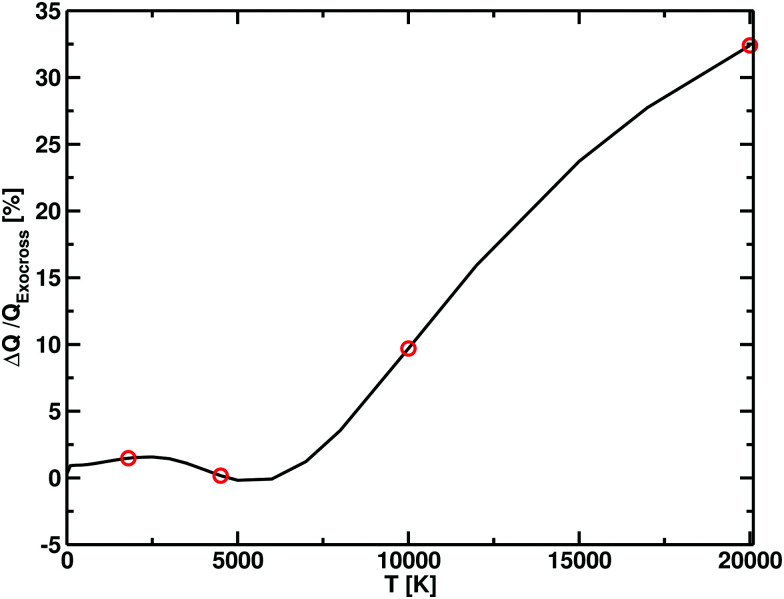
Difference in partition functions between Xu *et al.*^55^ and this work (Δ*Q*) as function of temperature, expressed in terms of percentage with respect to our (ExoCross) values. For temperatures below 7500 K, the difference between the two models is within the 2%, which increases at higher temperatures, with our model having higher values. The red dot points show the temperatures for which the photodissociation cross section are simulated.

**Fig. 9:**
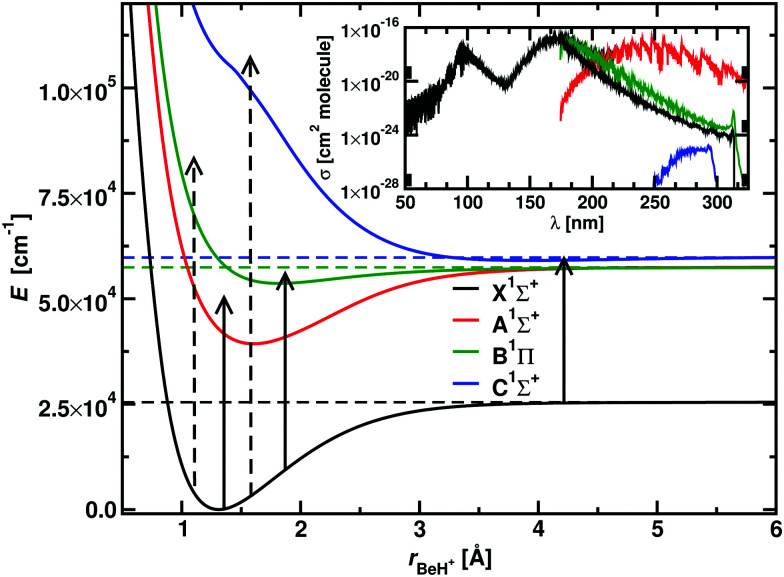
The main plot shows the BeH^+^ PECs from Xu *et al.*^55^ The solid vertical arrows show examples of bound–bound transitions, while the dashed vertical arrows represent bound–free transitions. An example of the photoabsorption spectrum at 1800 K is given in the inset: photodissociation is represented by the black curve, while each component of the bound–bound spectrum is represented by the colour of the final state in the main plot. A Gaussian smoothing model with HWHM = 75 cm^−1^ is used.

**Fig. 10:**
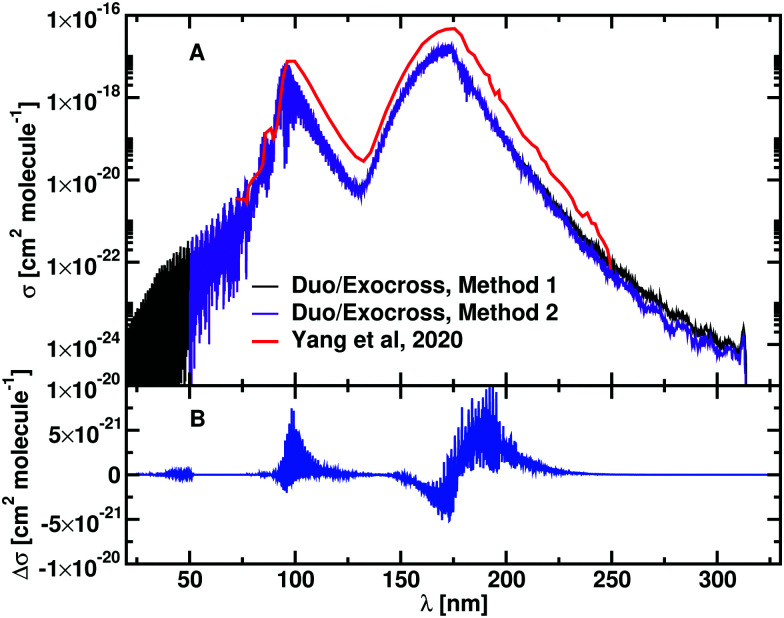
Panel A: photodissociation cross sections of BeH^+^ calculated at *T* = 1800 K, with two different approaches: in method 1, the complete photoabsorption spectrum is calculated and then the bound contributions are removed; in method 2 the single state photodissociation cross sections are calculated separately and then they are summed together. Panel B: the difference between method 1 and method 2 is plotted. Data from Yang *et al.*^56^ are plotted in red. A Gaussian smoothing model with HWHM = 75 cm^− 1^ is used.

**Fig. 11:**
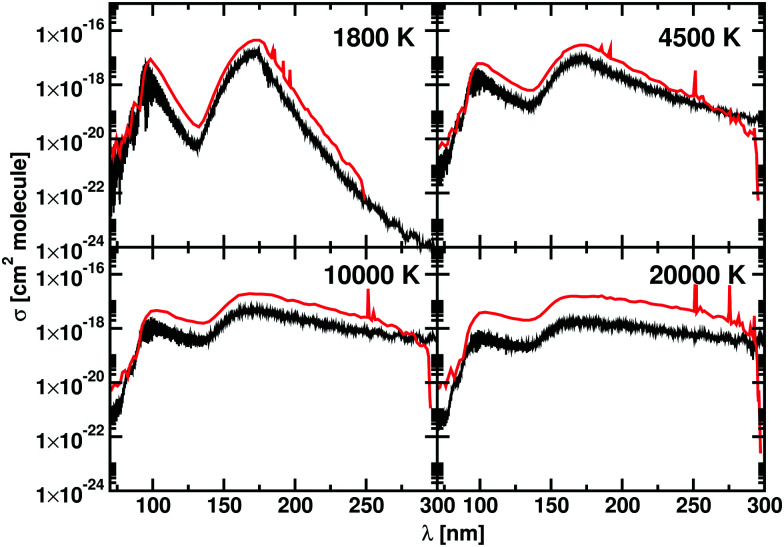
Photodissociation cross sections for BeH^+^: our calculations are in black, the data from Yang *et al.*^56^ are in red. Each panel shows a different temperature: 1800 K, 4500 K, 10 000 K, and 20 000 K. A Gaussian smoothing model with HWHM = 75 cm^−1^ is used.

The photodissociation cross sections form two peaks with maxima at *λ* = 172.31 nm and *λ* = 96.01 nm. As the temperature increases, the height of the two peaks decreases alongside a flattening of the regions between the cross sections. In Fig. 9, our computed spectra (black) are compared with the values of Yang *et al.*^56^ (red) for *T* = 1800 K, 4500 K, 10 000 K and 20 000 K. The photodissociation spectral contributions are obtained by subtracting the bound–bound components from the total photoabsorption spectrum, as shown in Fig. 9. The shapes and positions of the cross sections are the same in Yang *et al.*^56^ and our model, but our model consistently gives lower cross sections (Fig. 11). This discrepancy is directly proportional to the temperature. An important contribution to the discrepancy between our results and those of Yang *et al.*^56^ is the differences in the partition functions used. This difference is small for temperatures below 4500 K, where the two partition sums differ by about the 2% but is important for 10 000 K, where our partition function 10% bigger, and for 20 000 K, where our partition function is 30% bigger. Fig. 8 shows the difference of partition functions as function of the temperature; we believe ours to be more accurate (Fig. 11).

## 4 Conclusions

In this paper we develop a methodology which is suitable for the calculation of temperature-dependent photodissociation cross sections for diatomic molecules of arbitrary complexity. The methodology involves a solution of the coupled system of Schrödinger equations using bound vibrational basis functions and construction of pseudo-bound, temperature dependent cross-sections. For pure continuum cases, the photodissociation cross sections are generated using one of our smoothing approaches (Gaussian broadening or interpolation scheme). For hybrid, bound/free spectra, we use a stabilization method with averaged cross sections. We have tested the algorithm on a number of systems (HCl, HF, NaCl and BeH^+^) containing both pure repulsive excited state potential curves and ones which support bound states. Agreement, within understandable limits, is found between published data and our calculations for all molecules examined. Our plan is to include theoretical temperature-dependent photodissociation cross sections of HCl, HF, NaCl and BeH^+^ into the ExoMol data base, as part of the ExoMol project.^11^ Results of these studies, after some improvements of the corresponding spectroscopic models, will be published elsewhere.

A major motivation for the developments presented here is the need to provide temperature-dependent photodissociation cross sections for polyatomic molecules such water and CO_2_. We note that the DVR3D program suite^57^ has already been extended to provide rovibronic dipole transition intensities^58^ and to use Lobatto shape functions^59^ meaning that many of the developments for exploiting our proposed procedure are in place. However, for systems with more than two atoms, the identification of dissociative coordinates also becomes important.^59^ We plan to extend our calculations of photodissociation cross sections and rates to triatomic and larger molecules. This will require us to develop a rigorous procedure appropriate for multichannel systems.

## Data availability

Working examples of the input files used for generating the photodissociation cross sections and rates, together with the analysis tool are available as ESI† in the ESI.zip archive. A working version of Duo and ExoCross, which can be downloaded from the ExoMol github area, are required in order to run the input files. The file analysis.ipyn can be opened using Jupyter Notebook.

The directory duo-input contains the following input files that can be run using Duo, in order to create the initial state and transition files:

1 HCl-X-A.com, HF-X-A.com: input files for the A^1^Π← X^1^Σ^+^ band.

2 NaCl-Duo.com: input file for the A^1^Π ← X^1^Σ^+^ and the B^1^Σ^+^ ← X^1^Σ^+^s bands.

3 BeHp-X-A-Zhang.com, BeHp-X-B-Zhang.comBeHp-X-C-Zhang.com.

The directory analysis contains the files required for calculating the cross sections and photodissociation rates:

1 *hcl-2000.0.states* and *hcl-2000.0.trans*: output from DUO calculation of HCl.

2 *xsec-T2000.0.com*: ExoCross input file needed for generating the raw absorption cross sections at *T* = 2000 K assuming the Gaussian profile of HWHM = 10 cm^− 1^.

3 hcl-2000.0.xsec: output from *xsec-T2000.0.com*.

4 analysis.ipyn: analysis script for recovering the smoothed photodissociation cross section and the ISRF rates. All smoothing processes are included.

## Conflicts of interest

There are no conflicts to declare.

## Supplementary Material

CP-023-D1CP02162A-s001

CP-023-D1CP02162A-s002

CP-023-D1CP02162A-s003

CP-023-D1CP02162A-s004

CP-023-D1CP02162A-s005

CP-023-D1CP02162A-s006

CP-023-D1CP02162A-s007

CP-023-D1CP02162A-s008

CP-023-D1CP02162A-s009

CP-023-D1CP02162A-s010

CP-023-D1CP02162A-s011

CP-023-D1CP02162A-s013

## References

[cit1] Venot O., Rocchetto M., Carl S., Hashim A. R., Decin L. (2016). Astrophys. J..

[cit2] Badhan M. A., Wolf E. T., Kopparapu R. K., Arney G., Kempton E. M.-R., Deming D., Domagal-Goldman S. D. (2019). Astrophys. J..

[cit3] Fleury B., Gudipati M. S., Henderson B. L., Swain M. (2019). Astrophys. J..

[cit4] Lewis N. K., Wakeford H. R., MacDonald R. J., Goyal J. M., Sing D. K., Barstow J., Powell D., Kataria T., Mishra I., Marley M. S., Batalha N. E., Moses J. I., Gao P., Wilson T. J., Chubb K. L., Mikal-Evans T., Nikolov N., Pirzkal N., Spake J. J., Stevenson K. B., Valenti J., Zhang X. (2020). Astrophys. J..

[cit5] Valiev R., Berezhnoy A., Gritsenko I., Merzlikin B., Cherepanov V., Kurten T., Wöhler C. (2020). Astron. Astrophys..

[cit6] Heays A. N., Bosman A. D., van Dishoeck E. F. (2017). Astron. Astrophys..

[cit7] Noelle A., Vandaele A. C., Martin-Torres J., Yuan C., Rajasekhar B. N., Fahr A., Hartmann G. K., Lary D., Lee Y.-P., Limao-Vieira P., Locht R., McNeill K., Orlando J. J., Salama F., Wayne R. P. (2020). J. Quant. Spectrosc. Radiat. Transfer.

[cit8] Schinke R. , Photodissociation Dynamics, Cambridge University Press, 1993

[cit9] Grebenshchikov S. Y. (2016). J. CO2 Util..

[cit10] Tennyson J., Yurchenko S. N. (2012). Mon. Not. R. Astron. Soc..

[cit11] Tennyson J., Yurchenko S. N., Al-Refaie A. F., Clark V. H. J., Chubb K. L., Conway E. K., Dewan A., Gorman M. N., Hill C., Lynas-Gray A. E., Mellor T., McKemmish L. K., Owens A., Polyansky O. L., Semenov M., Somogyi W., Tinetti G., Upadhyay A., Waldmann I., Wang Y., Wright S., Yurchenko O. P. (2020). J. Quant. Spectrosc. Radiat. Transfer.

[cit12] Tennyson J., Yurchenko S. N. (2017). Int. J. Quantum Chem..

[cit13] Yurchenko S. N., Lodi L., Tennyson J., Stolyarov A. V. (2016). Comput. Phys. Commun..

[cit14] Tennyson J., Lodi L., McKemmish L. K., Yurchenko S. N. (2016). J. Phys. B: At., Mol. Opt. Phys..

[cit15] Qu Q., Cooper B., Yurchenko S. N., Tennyson J. (2021). J. Chem. Phys..

[cit16] Rivlin T., McKemmish L. K., Spinlove K. E., Tennyson J. (2019). Mol. Phys..

[cit17] Patrascu A. T., Tennyson J., Yurchenko S. N. (2015). Mon. Not. R. Astron. Soc..

[cit18] Yurchenko S. N., Blissett A., Asari U., Vasilios M., Hill C., Tennyson J. (2016). Mon. Not. R. Astron. Soc..

[cit19] Yurchenko S. N., Szabo I., Pyatenko E., Tennyson J. (2018). Mon. Not. R. Astron. Soc..

[cit20] McKemmish L. K., Masseron T., Hoeijmakers J., Pérez-Mesa V. V., Grimm S. L., Yurchenko S. N., Tennyson J. (2019). Mon. Not. R. Astron. Soc..

[cit21] Yurchenko S. N., Smirnov A. N., Solomonik V. G., Tennyson J. (2019). Phys. Chem. Chem. Phys..

[cit22] Qu Q., Yurchenko S. N., Tennyson J. (2021). Mon. Not. R. Astron. Soc..

[cit23] Yurchenko S. N., Al-Refaie A. F., Tennyson J. (2018). Astron. Astrophys..

[cit24] Colbert D. T., Miller W. H. (1992). J. Chem. Phys..

[cit25] Manolopoulos D. E. , Numerical Grid Methods and Their Application to Schrödinge's Equation, Springer, 1993, pp. 57–68

[cit26] Le Roy R. J., Macdonald R. G., Burns G. (1976). J. Chem. Phys..

[cit27] Virtanen P., Gommers R., Oliphant T. E., Haberland M., Reddy T., Cournapeau D., Burovski E., Peterson P., Weckesser W., Bright J., van der Walt S. J., Brett M., Wilson J., Millman K. J., Mayorov N., Nelson A. R. J., Jones E., Kern R., Larson E., Carey C. J., Polat İ., Feng Y., Moore E. W., VanderPlas J., Laxalde D., Perktold J., Cimrman R., Henriksen I., Quintero E. A., Harris C. R., Archibald A. M., Ribeiro A. H., Pedregosa F., van Mulbregt P., SciPy 1.0 Contributors (2020). Nat. Methods.

[cit28] Hill C., Yurchenko S. N., Tennyson J. (2013). Icarus.

[cit29] Hazi A. U., Taylor H. S. (1970). Phys. Rev. A: At., Mol., Opt. Phys..

[cit30] Mandelshtam V. A., Ravuri T. R., Taylor H. S. (1993). Phys. Rev. Lett..

[cit31] Bacic Z., Simons J. (1982). J. Phys. Chem..

[cit32] Wakelam V., Herbst E., Loison J.-C., Smith I. W. M., Chandrasekaran V., Pavone B., Adams N. G., Bacchus-Montabonel M.-C., Bergeat A., Béroff K., Bierbaum V. M., Chabot M., Dalgarno A., van Dishoeck E. F., Faure A., Geppert W. D., Gerlich D., Galli D., Hebrard E., Hersant F., Hickson K. M., Honvault P., Klippenstein S. J., Le Picard S., Nyman G., Pernot P., Schlemmer S., Selsis F., Sims I. R., Talbi D., Tennyson J., Troe J., Wester R., Wiesenfeld L. (2012). Astrophys. J., Suppl. Ser..

[cit33] Wakelam V., Loison J.-C., Herbst E., Pavone B., Bergeat A., Béroff K., Chabot M., Geppert A. F. W. D., Gerlich D., Gratier P., Harada N., Hickson K. M., Honvault P., Klippenstein S. J., Le Picard S., Nyman G., Ruaud M., Schlemmer S., Sims I. R., Talbi D., Tennyson J., Wester R. (2015). Astrophys. J., Suppl. Ser..

[cit34] Draine B. T. (1978). Astrophys. J., Suppl. Ser..

[cit35] van Dishoeck E. F., Black J. H. (1982). Astrophys. J..

[cit36] Lee L. C. (1984). Astrophys. J..

[cit37] Alexander M. H., Pouilly B., Duhoo T. (1993). J. Chem. Phys..

[cit38] Givertz S. C., Balint-Kurti G. G. (1986). J. Chem. Soc., Faraday Trans..

[cit39] Brown A., Balint-Kurti G. G. (2000). J. Chem. Phys..

[cit40] Balint-Kurti G. G., Mort S. P., Marston C. C. (1993). Comput. Phys. Commun..

[cit41] Inn E. C. Y. (1975). J. Atmos. Sci..

[cit42] Nee J. B., Suto M., Lee L. C. (1986). J. Chem. Phys..

[cit43] Cheng B.-M., Chung C.-Y., Bahou M., Lee Y.-P., Lee L. C. (2002). J. Chem. Phys..

[cit44] van Dishoeck E. F., van Hemert M. C., Dalgarno A. (1982). J. Chem. Phys..

[cit45] Hitchcock A. P., Williams G. R. J., Brion C. E., Langhoff P. W. (1984). Chem. Phys..

[cit46] Nee J. B., Suto M., Lee L. C. (1985). J. Phys. B: At., Mol. Opt. Phys..

[cit47] Silver J. A., Worsnop D. R., Freedman A., Kolb C. E. (1986). J. Chem. Phys..

[cit48] Davidovits P., Brodhead D. C. (1967). J. Chem. Phys..

[cit49] Barton E. J., Chiu C., Golpayegani S., Yurchenko S. N., Tennyson J., Frohman D. J., Bernath P. F. (2014). Mon. Not. R. Astron. Soc..

[cit50] Werner H. J. , Knowles P. J., Lindh R., Manby F. R. and Schütz M., MOLPRO, a package of ab initio programs, 2010, see http://www.molpro.net/

[cit51] Primas F., Molaro P., Bonifacio P., Hill V. (2000). Astron. Astrophys..

[cit52] Pinsonneault M., Kawaler S. D., Demarque P. (1990). Astrophys. J., Suppl. Ser..

[cit53] Celiberto R., Janev R. K., Reiter D. (2012). Plasma Phys. Controlled Fusion.

[cit54] Laporta V., Chakrabarti K., Celiberto R., Janev R. K., Mezei J. Z., Niyonzima S., Tennyson J., Schneider I. F. (2017). Plasma Phys. Controlled Fusion.

[cit55] Xu X.-S., Dai A.-Q., Peng Y.-G., Wu Y., Wang J.-G. (2018). J. Quant. Spectrosc. Radiat. Transfer.

[cit56] Yang Y. K., Cheng Y., Peng Y. G., Wu Y., Wang J. G., Qu Y. Z., Zhang S. B. (2020). J. Quant. Spectrosc. Radiat. Transfer.

[cit57] Tennyson J., Kostin M. A., Barletta P., Harris G. J., Polyansky O. L., Ramanlal J., Zobov N. F. (2004). Comput. Phys. Commun..

[cit58] Zak E. J., Tennyson J. (2017). J. Chem. Phys..

[cit59] McKemmish L. K., Tennyson J. (2019). Philos. Trans. R. Soc., A.

